# Epiphytic fungi induced pathogen resistance of invasive plant *Ipomoea cairica* against *Colletotrichum gloeosporioides*

**DOI:** 10.7717/peerj.8889

**Published:** 2020-04-13

**Authors:** Hua Xu, Minjie Zhu, Shaoshan Li, Weibin Ruan, Can Xie

**Affiliations:** 1 Key Laboratory of Ecology and Environmental Science in Guangdong Higher Education, School of Life Science, South China Normal University, Guangzhou, China; 2 Department of Biotechnology, Beijing Normal University Zhuhai Campus, Zhuhai, China; 3 College of Life Sciences, Nankai University, Tianjin, China

**Keywords:** Symbiosis, Non-pathogen, NPR1 gene, Hormone, β-1,3-glucanase, Signaling molecules, Hydrogen peroxide

## Abstract

**Background:**

*Ipomoea cairica* (L.) Sweet is a destructive invasive weed in South China but rarely infected with pathogens in nature. Its pathogen resistance mechanism is largely unknown at present. Some non-pathogenic isolates of *Fusarium oxysporum* and *Fusarium fujikuroi* are prevalent on many plant species and function as pathogen resistance inducers of host plants. The objective of the present research is to investigate whether the symbiosis between the both fungi and *I. cairica* is present, and thereby induces pathogen resistance of *I. cairica.*

**Methods:**

Through field investigation, we explored the occurrence rates of *F. oxysporum* and *F. fujikuroi* on leaf surfaces of *I. cairica* plants in natural habitats and compared their abundance between healthy leaves and leaves infected with *Colletotrichum gloeosporioides*, a natural pathogen. With artificial inoculation, we assessed their pathogenicity to *I. cairica* and studied their contribution of pathogen resistance to *I. cairica* against *C. gloeosporioides.*

**Results:**

We found that *F. oxysporum* and *F. fujikuroi* were widely epiphytic on healthy leaf surfaces of *I. cairica* in sunny non-saline*,* shady non-saline and sunny saline habitats. Their occurrence rates reached up to 100%. Moreover, we found that the abundance of *F. oxysporum* and *F. fujikuroi* on leaves infected with *C. gloeosporioides* were significantly lower than that of healthy leaves. With artificial inoculation, we empirically confirmed that *F. oxysporum* and *F. fujikuroi* were non-pathogenic to *I. cairica*. It was interesting that colonization by* F. fujikuroi*, *F. oxysporum* alone and a mixture of both fungi resulted in a reduction of *C. gloeosporioides* infection to *I. cairica* accompanied by lower lesion area to leaf surface area ratio, increased hydrogen peroxide (H_2_O_2_) concentration and salicylic acid (SA) level relative to the control. However, *NPR1* expression, chitinase and β-1,3-glucanase activities as well as stem length and biomass of *I. cairica* plant only could be significantly improved by *F. oxysporum* and a mixture of both fungi but not by *F. fujikuroi*. In addition, as compared to colonization by *F. oxysporum* and a mixture of both fungi,* F. fujikuroi* induced significantly higher jasmonic acid (JA) level but significantly lower β-1,3-glucanase activity in leaves of *I. cairica* plants. Thus, our findings indicated the symbiosis of epiphytic fungi*****F. fujikuroi* and *F. oxysporum* induced systemic resistance of *I. cairica* against* C. gloeosporioides*. *F. oxysporum* played a dominant role in inducing pathogen resistance of *I. cairica*. Its presence alleviated the antagonism of the JA signaling on SA-dependent β-1,3-glucanase activity and enabled* I. cairica* plants to maintain relatively higher level of resistance against *C. gloeosporioides.*

## Introduction

Fungal epiphytes are a group of microbes which colonize the surface of the plants and establish various relationships with their hosts. These associations range from epiphytic commensals, mutualistic symbionts to pathogens (Kowalski et al., 2015). *Fusarium oxysporum* and *Fusarium fujikuroi* are polytypic species complex with anamorphs in *Fusarium,* which are prevalent on the leaf, stem, root, seed and inflorescence surfaces of many economically-important plants such as *Ananas comosus* (Dianese et al., 1981)*, Ipomoea batatas* (Clark, Hoy & Nelson, 1995) and *Oryza sativa* (Choi et al., 2018)*.* Some isolates within *F. fujikuroi* or *F*. *oxysporum* species can trigger gibberellin-induced bakanae disease of *O. sativa* (Hwang et al., 2013), pitch canker of *Pinus* spp. (Herron et al., 2015), stalk rot of *Zea mays* and *Sorghum bicolor* (Leslie, 1995), stem wilt and root rot of *Schlumbergera truncata* (Lops et al., 2013) and crown disease of oil palm (Hafizi, Salleh & Latiffah, 2013). However, it was reported that the isolates of *F. fujikuroi* and *F*. *oxysporum* were entirely nonpathogenic and avirulent to their hosts, such as *A. comosus* (Dianese et al., 1981), *O. sativa* (Choi et al., 2018; Amatulli et al., 2010) and *Glycine max* (Lanubile et al., 2015). It is known that many pathogenic and nonpathogenic *F. oxysporum* or *F. fujikuroi* isolates elicited the systemic acquired resistance (SAR) or induced systemic resistance (ISR) of their plant hosts to confer resistance against a broad spectrum of pathogens (Paparu et al., 2007; Patil et al., 2011; Veloso & Díaz, 2012; Matić et al., 2016; Miyaji et al., 2017). These *Fusarium* spp. studied earlier are plant endophytes or soil-borne fungi. Epiphytic *F. oxysporum* or *F. fujikuroi* involving ISR and SAR of plants to date is little known. Pathogen resistance is induced through the accumulation of salicylic acid (SA) or jasmonic acid (JA) (Mandal, Mallick & Mitra, 2009; Chen et al., 2018; Jin et al., 2018) and the expression of non-expressor of pathogenesis-related genes-1 (*NPR1*) as well as pathogenesis-related (PR) proteins (Stein et al., 2008; Nic-Matos et al., 2017; Ali et al., 2017). Cytosolic hydrolytic enzymes such as β-1,3-glucanases and chitinases are members of PR1 proteins (Fagoaga et al., 2001; Park et al., 2004), and exert inhibitory effects on the fungal growth through degrading chitin and glucan in the cell wall of pathogenic fungi (Balasubramanian et al., 2012; Vieira et al., 2010). In addition, the accumulation of reactive oxygen species (ROS) such as hydrogen peroxide (H_2_O_2_) is frequently involved in the defense responses, which may kill pathogens directly (Lin & Ishii, 2009).

*Ipomoea cairica* (L.) Sweet is native to tropical Africa and is causing a serious invasive ecological problem in South China (Huang et al., 2009). This weed usually occurs in non-arable lands, wastelands, forests edges and farmlands where it invades the original diverse community which is always reduced to a monoculture (Li et al., 2012). Owing to its strong salt tolerance, *I. cairica* has been found to have successfully invaded into mangrove wetland in the coastal areas (Liu et al., 2012; Liu et al., 2016) and seriously threatened local eco-systematic functions. In addition, *I*. *cairica* has strong pathogen resistance and is scarcely infected with pathogens in nature. Only a disease symptom caused by *Colletotrichum gloeosporioides* was observed sporadically on *I. cairica* plants in previous field investigations (Lin & Liu, 2010). Although several herbicides have shown significant efficacies in controlling *I*. *cairica*, their application may lead to environmental pollution (Sun et al., 2015). Thus, the use of biological control is an attractive option. To help with the biological control of *I. cairica*, it is important to understand its mechanisms of pathogen resistance which to date remain relatively unknown.

Considering the omnipresence of *F. oxysporum* and *F. fujikuroi* on the surface of plants in natural surroundings and their roles in inducing plant pathogen resistance, we hypothesized that the occurrence of *F*. *oxysporum* or *F*. *fujikuroi* on leaves might be involved in the antagonistic character of *I. cairica* against plant pathogen. The objectives of the present study were therefore to elucidate the following questions: (i) whether *F*. *oxysporum* or *F*. *fujikuroi* widely occur on the leaf surface of *I. cairica* in the field; (ii) whether there is different abundance of *F. oxysporum* or *F. fujikuroi* between leaves infected with *C. gloeosporioides* and healthy leaves; (iii) if the former two questions are positive then whether *F. oxysporum* or *F. fujikuroi* can induce the pathogen resistance of *I. cairica* against *C. gloeosporioides.*

## Materials & Methods

### The occurrence rate and abundance of *F. oxysporum* and *F. fujikuroi* on leaves of *I. cairica* in the field

To investigate the occurrence of epiphytic *F. oxysporum* and *F. fujikuroi*, sunny saline, sunny non-saline and shady non-saline habitats with *I. cairica* were selected for leaf sampling. The saline habitat was located in intertidal zones of Yakou village, Zhongshan city (22°28′1.03″N, 113°32′42.56″E). The non-saline habitat was located in Huitong village, Zhuhai city (22°21′26.60″N, 113°30′46.18″E). The linear distance between the saline and non-saline habitats is about 20 km. In each habitat, five *I. cairica* populations covering more than 50 m^2^ were selected as sample plots. Distances between sample plots are more than 500 m. In each sample plot, three sample sites covering about 4 m^2^ were selected randomly. In each sample site, three healthy leaves of *I. cairica* were excised and pooled together as a sample, then stored in a sterile plastic Ziploc^®^ (Racine, WI) bag and returned to the laboratory. A total of 45 samples (3 habitats ×5 sample plots ×3 sample sites) were used for analyzing the occurrence rate of *F. oxysporum* and *F. fujikuroi*.

Fungal epiphytes were isolated from the leaves of *I. cairica* following the method of Salazar-Cerezo et al. (2018). Briefly, each sample was dipped into 75 ml of sterile distilled water contained in a 250 ml sterile conical flask. The conical flask was set on a shaker (IKA, Staufen, Germany) at 170 rpm for 60 min. The resulting suspension (200 µl) was plated onto sterile potato dextrose agar (PDA) contained in a sterile Petri dish. The plating was performed in triplicate. Control treatments only contained sterile water and PDA. All plates were incubated in dark at 26 °C for 15 days. Plates were checked daily and each emerging fungal colony was transferred onto a fresh PDA until axenic cultures were obtained. These cultures were classified into diverse morphotypes according to color, texture and colony morphology, and were used for species identification. The value 1 and 0 indicated the presence or absence of *F. oxysporum* and *F. fujikuroi*, respectively on each sample. These values were used to assess the occurrence of *F. oxysporum* and *F. fujikuroi*.

In addition, the abundance of *F. oxysporum* and *F. fujikuroi* on *I. cairica* in the field was compared between healthy leaves and leaves infected with *C. gloeosporioides*. In Huitong village, Zhuhai city, three sample plots covering more than 100 m^2^ were selected for leaf sampling. Six healthy leaves and equal number of infected leaves of *I. cairica* were collected from each sample plot and separately stored in a sterile plastic Ziploc^®^ bag as a healthy and infected sample, then returned to the laboratory for further analyses. After weighing of each sample, fungal isolation, purification and morphotype classification were performed using the above methods. The colony-forming units (CFU) of *F. oxysporum* and *F. fujikuroi* were recorded. The abundance of *F. oxysporum* and *F. fujikuroi* was expressed as CFU per gram fresh weight (FW).

### Fungal identification

The internal transcribed spacer (ITS) sequences of nuclear ribosomal DNA (rDNA) have been successfully used in resolving phylogenetic relationships of the fungi in the genera *Fusarium* (Lin et al., 2014) and *Colletotrichum* (Weir, Johnston & Damm, 2012). Combining with morphological characteristics, these fungi can be identified well to species level (Kvas et al., 2009; Lin et al., 2014; Rabha et al., 2016; Moya-Elizondo et al., 2019). In this work, the identification of *F. fujikuroi* and *F. oxysporum* along with a natural pathogen of *I. cairica*, *C. gloeosporioides* was performed with a combined method of molecular and morphological analyses. *C. gloeosporioides* was isolated directly from leaves of *I. cairica* infected with *C. gloeosporioides* in the field*.* Briefly, a leaf spot was excised from infected leaf and transferred on sterile PDA contained in a sterile Petri dish, and then incubated in dark at 26 °C. Subsequent process of fungal purification was performed using the above method*.* According to the differentiation of colony morphology, the cultures of these three fungi were divided into three morphotypes.

Three representative isolates (JY1, JY2 and JY3) were selected from their respective related morphotypes, and were used for analyzing the ITS sequences. Genomic DNA was extracted from 0.1 g of mycelia using CTAB method (Séne et al., 2015). *Following the methods of Lin et al. (2014) and* Rabha et al. (2016), PCR amplification of the ITS region was performed using fungus-specific primers ITS1-F (5′-CTTGGTCATTTAGAGGAAGTAA-3′) and ITS4-R (5′-TCCTCCGCTTATTGATATGC-3′). PCR amplification reactions were conducted with 10 ng of template DNA, 0.5 µM of each primer, 17.5 µl of the Premix *Taq* (TaKaRa, Dalian, China) and double distilled water (ddH_2_O) in a final volume of 35 µl. The program used for PCR was as follows: 95 °C for 5 min (1 cycle); 95 °C for 30 s, 51.6 °C for 45 s, 72 °C for 90 s (30 cycles); 72 °C for 7 min (1 cycle). Amplification products were sequenced using the services provided by Sangon Biotech Co., Ltd. (Shanghai, China). These sequences were submitted to GenBank under accession numbers MN704851.1, MN704852.1 and MN704853.1, and were compared against those sequences published in GenBank using the BLAST search program (http://www.ncbi.nlm.nih.gov/BLAST). The sequences of related fungi were obtained from GenBank as follows: *Colletotrichum siamense* (MN296060.1, MN296066.1 and KP635210.1), *C. gloeosporioides* (JQ936115.1, MF314168.1 and MH930419.1), *F. oxysporum* (FJ867936.1, KY305290.1 and MK156682.1) and *F. fujikuroi* (KT192276.1, KP998524.1 and LS422781.1). All of sequences were aligned with using CLUSTAL W (Thompson, Higgins & Gibson, 1994) present in MEGA 5 software (Tamura et al., 2011). Aligned sequences were used to construct phylogenetic tree using the neighbor-joining (NJ) and Kimura 2-parameter methods (Saitou & Nei, 1987; Kimura, 1980). Bootstrap resampling (1,000 replications) was used as a statistical support for nodes in the phylogenetic tree. *Penicillium oxalicum* (MH634489.1) was used as an outgroup.

Fourteen days old fungal cultures were used for morphological identification of *F. fujikuroi* and *F. oxysporum* according to the *Fusarium* Laboratory Manual (Leslie & Summerell, 2006). Morphological analysis of *C. gloeosporioides* was performed following the method of Rabha et al. (2016). Morphological characteristics including conidial length, width and septation were measured in a photonic microscope (Nikon, Tokyo, Japan).

### Pathogenicity identification of *F. oxysporum* and *F. fujikuroi*

In order to identify the pathogenicity of *F. oxysporum* and *F. fujikuroi* to *I. cairica*, with artificial inoculation, the lesion areas caused by the two fungi were compared with a positive control and a negative control. The positive control was inoculated with *C. gloeosporioides.* The negative control was sprayed with sterile potato dextrose broth (Huankai Co., Ltd., Guangzhou, China).

### Plant materials

Two hundred cuttings (10 cm length, three mm diameter) each with two healthy leaves were clipped from an *I. cairica* population in the field in Huitong village, Zhuhai city. These cuttings were cultivated with sterile Hoagland nutrient solution for a week.

### Fungi materials

Fungal isolates identified as *F. oxysporum*, *F. fujikuroi* and *C. gloeosporioides* in the previous experiments were subcultivated on fresh PDA and used as experiment materials. Twenty days old cultures of *F. oxysporum*, *F. fujikuroi* and *C. gloeosporioides* were used for preparation of their respective conidial suspensions. Briefly, the mycelia were transferred to 200 ml of sterile potato dextrose broth contained in a 250 ml sterile conical flask, which was then sealed with parafilm, shaken repeatedly and incubated in dark at 26 °C. After 24 h, the fungal suspension was filtered with three layers of sterile gauze to obtain the conidial suspension. Conidial concentration of each fungal species was determined using hemocytometer (Shanghai Medical Optical Instrument Plant, Shanghai, China) and photonic microscope, then adjusted to 1 × 10^7^ ml^−1^ with sterile potato dextrose liquid broth according to the methods of Matić et al. (2016) and Aimé et al. (2008) with some modification.

### Pathogenicity assessment

Thirty two cuttings of *I. cairica* were selected for assessing pathogenicities of *F. oxysporum*, *F. fujikuroi* and *C. gloeosporioides*. The leaf surface was sterilized by cleaning twice with the degreased cotton immersed by 75% (v/v) ethanol. These cuttings were divided evenly into four groups including three treatments and one control. Thus, each treatment was repeated eight times. One hour later, the three treatments were inoculated with conidial suspension of *F. oxysporum*, *F. fujikuroi* and *C. gloeosporioides*, respectively. Inoculation volume of conidial suspension on each leaf was 2.5 ml. Leaves in the control group were sprayed with an equal volume of sterile potato dextrose broth. All inoculated cuttings were then cultivated with sterile Hoagland nutrient solution in sterile illuminating incubator at 28 ± 1 °C with a 14/10 h photoperiod (cool- white neon tube (200 µmol m^−2^ s^−1^). The relative humidity in the illuminating incubator was maintained at 75%–80%. After 7 days, all cuttings were harvested, and then the inoculated leaves of each cutting were photographed with a digital camera. Total number of pixel of lesions and pixels of the whole leaf were measured with Adobe Photoshop CS6 software (Adobe System Inc., San Jose, CA, USA). The lesion area ratio was calculated as the % of the whole leaf area.

### Plant pathogen resistance induced by *F. oxysporum*, *F. fujikuroi* alone and in a mixture

One hundred and twenty cuttings of *I. cairica* were divided evenly into four groups including three treatments and one control. The leaf surface was sterilized as above. One hour later, three treatments were pre-inoculated with the conidial suspension of *F. oxysporum*, *F. fujikuroi* alone or a mixture of both fungi, respectively. The conidial suspension of the fungal mixture of the two species was prepared with an equal volume of conidial suspension of *F. oxysporum* and *F. fujikuroi*. Pre-inoculation volume of conidial suspension on each leaf was 2.5 ml. The leaves in the control group were sprayed with an equal volume of sterile potato dextrose broth. All cuttings were then cultivated with sterile Hoagland nutrient solution in sterile illuminating incubator at 28 ± 1 °C with a 14/10 h photoperiod (cool- white neon tube (200 µmol m^−2^ s^−1^). The relative humidity in the illuminating incubator was maintained at 75%–80%. After 3 days, these cuttings were removed and inoculated with 2.5 ml of conidial suspension of *C. gloeosporioides* per leaf, then returned to illuminating incubator. After 15 days, these cuttings were harvested.

### Growth parameter determination: Stem length, biomass and lesion area ratio

The stem length of five random replicates was measured from the harvested cuttings of each treatment and the control group. Each cutting was then clipped into small pieces and dried at 75 °C in a drying oven for 10 h to weigh the biomass. Five random replicates of harvested cuttings from each treatment and the control were sampled to determine the lesion area ratio according to the method described above.

### Physiological characteristics measurement

Four random replicates of the harvested cuttings in each treatment and the control group were sampled to determine H_2_O_2_ concentration, β-1,3-glucanase and chitinase activity. The leaf was excised from each cutting of *I*. *cairica*, deveined and stored at −80 °C.

H_2_O_2_ was extracted according to the method of Ferguson, Watkins & Harman (1983). The deveined leaf (0.2 g FW) was homogenized in 5 ml cold acetone in a mortal. The extract and washings were centrifuged (4,000 rpm) at 4 °C for 10 min. The supernatant was used to measure H_2_O_2_ concentration by modification of the method of Brennan & Frenkel (1977). One milliliter of the supernatant was added to 250 µl of 50 mg ml^−1^ Ti(SO_4_)_2_ in concentrated H_2_SO_4_. The solution was shaken, followed by the addition of 2 ml concentrated NH_4_OH and thoroughly mixed. After centrifugation (20 min at 4,000 rpm), the supernatant was discarded and the precipitate washed repeatedly with 4 ml acetone until the supernatant was colorless. The precipitate was solubilized in 4 ml 2 NH_2_SO_4_. The absorbance of the obtained solutions was recorded at 415 nm against a water blank. The concentration of H_2_O_2_ in the extracts was determined by comparing the absorbance against a standard curve representing 0–80 µmol ml^−1^H_2_O_2_.

The extraction of β-1,3-glucanase and chitinase was based on the method of Magnin­ Robert et al. (2007). Deveined leaf (0.2 g FW) was homogenized in 5 ml cold sodium acetate buffer, PH 5.0 containing one mmol dithiothreitol and 10 mg phenylmethysulfonyl fluoride in a cold mortal. The crude extracts were centrifuged at 4,000 rpm for 50 min at 4 °C and supernatants were used in enzymatic activity assays.

β-1,3-glucanase activity was measured according to the method of De la Cruz et al. (1995). The reaction was started by mixing 200 µl of the supernatant and 200 µl of laminarin (1 mg ml^−1^). The mixture was incubated at 37 °C for 30 min, followed by the addition of 2 ml of dinitrosalicylic acid (DNS) reagent (Sangon Biotech Co., Ltd., Shanghai, China), then boiled for 5 min. Enzyme and substrate blank were also included. The absorbance of the obtained solution was recorded at 600 nm. A standard curve was established with 0 to 80 mg ml^−1^ glucose. A unit of β-1,3-glucanase activity was defined as the amount of enzyme catalyzing the release of 1 µmol of glucose equivalent per minute.

Chitinase activity was measured according to the method of Chen & Lee (1994). The mixture containing 400 µl of the supernatant and 400 µl of colloidal chitin (10 mg ml^−1^) was incubated at 37 °C for 1 h, followed by the addition of 1.5 ml of DNS reagent, then boiled for 5 min. Enzyme and substrate blank were also included. The absorbance of the obtained solution was recorded at 530 nm. A standard curve was established with 0–1 mg ml^−1^ N-acetylglucosamine (NAG). A unit of chitinase activity was defined as the amount of enzyme catalyzing the release of 0.5 µmol of NAG equivalent per hour.

### Hormone measurement

Nine harvested cuttings in each treatment and the control group were sampled to determine SA and JA. Leaves of three cuttings in each treatment or the control group were cut into pieces and pooled together as a sample, stored at −80 °C. Thus, each treatment was repeated 3 times.

SA and JA were extracted following the method of Engelberth et al. (2003), with some modification. Frozen leaves (1.0 g) in each sample were weighed and ground in liquid nitrogen to a fine powder. Extraction was done by adding 10 ml of methanol and transferring the mixture to a 50 ml centrifuge tube, then set on a shaker at 300 rpm for 2 h. After centrifugation at 4,000 rpm for 5 min, the supernatant was transferred to another centrifuge tube and concentrated under a flow of nitrogen gas. The residue was reconstituted with 1 ml of methanol, then was filtered through a 0.2- µm-Teflon filter into an autosampler vials.

According to the method of Ratzinger et al. (2009), with some modification, an AB Sciex Qtrap^®^ 5500 LC/MS/MS system (AB Sciex, Foster City, CA, USA) with multiple reaction monitoring mode was used to quantify SA and JA. The sample was injected onto a reverse-phase column PAK C18-ARC (150 × 2.0 mm, 3 µm, Shiseido, Tokyo, Japan) kept at 25 °C and eluted isocratically with the mobile phase consisting of 5 mM ammonium acetate (mobile phase A) and acetonitrile (mobile phase B) at a flow rate of 0.3 ml min^−1^. The injection volume was 0.2 µl. The eluate was subjected to positive electrospray ionization, and the ions were detected using the following mass transitions: SA m/z 137.0 → m/z 93; JA m/z 209.0 → m/z 59.0.

The external standard working fluids for calibration curves were established with 2–100 ng ml^−1^ of SA and JA in methanol. The standards of SA and JA were purchased from ZZBIO Co., Ltd (Shanghai, China).

### Real-time RT-PCR analysis of *NPR1* expression

Three replicates of the harvested cuttings in each treatment and the control group were sampled to analyze *NPR1* expression. Total RNA was extracted from leaves using Total RNA Purification Reagent Kit (Sangon Biotech Co., Ltd., Shanghai, China) according to manufacturer’s instructions. First strand cDNA was synthesized from 1 µg of total RNA using Reverse Transcription System (DaAn Gene Co., Ltd., Guangzhou, China) according to the manufacturer’s instructions. Following the method of Aimé et al. (2008), the actin gene was used as a reference gene. Based on *NPR1* (accession numbers: EF190039.1, XM_019312052.1, XM_019317156.1 and XM_019317920.1) and actin gene (accession numbers: AY905538.1, GU395493.1, XM_019297139.1 and D78205.1) mRNA sequences of homogenous species *Ipomoea nil* and *Ipomoea batatas* deposited in GenBank, *NPR1* primers (5′-CTTCAGGAGCGTATTTAGTGG-3′and 5′- AAAACAGTCACTACGGCATCA-3′) and actin gene primers (5′- GCGGATAGAATGAGCAAGG-3′and 5′- GAGCCTCCAATCCAGACAC-3′) of *I. cairica* were designed respectively by Primer3 software (http://fokker.wi.mit.edu/primer3/input.htm).

Real-time PCR reactions were conducted with 10 ng of cDNA, 200 nM of each primer, 10 µl of the SYBR green master mix (TaKaRa, Dalian, China) and double distilled water (ddH_2_O) in a final volume of 20 µl. In the negative control, cDNA was replaced by ddH_2_O. Reactions were performed on an ABI PRISM 7500HT Sequence Detection System (Applied Biosystems, Foster City, CA, USA). The program used for real-time PCR was as follow: 10 s at 95 °C, 45 cycles of 5 s at 95 °C, 30 s at 53 °C and 34 s at 72 °C. Two replicates of real-time PCR reactions were performed for each sample.

The melting curve analysis was performed to verify the sensitivity and specificity of primers. After the real-time PCR finished, Ct number was extracted for both actin gene and *NPR1* gene with auto baseline and manual threshold. The relative expression of *NPR1* gene and actin gene were calculated according to the 2^−ΔCt^ method provided by Schmittgen & Livak (2008). Δ Ct = Ct_*NPR*1_ − Ct_actin_.

### Data analyses

Statistical analyses were performed on SPSS 16.0 software (IBM, Chicago, IL, USA) using one-way analysis of variance (ANOVA) followed by LSD’s post-hoc test. The values were expressed as the means ± standard errors and *P* values < 0.05 were considered statistically significant.

## Results

### The identification of representative isolates JY1, JY2 and JY3

**Figure 1:**
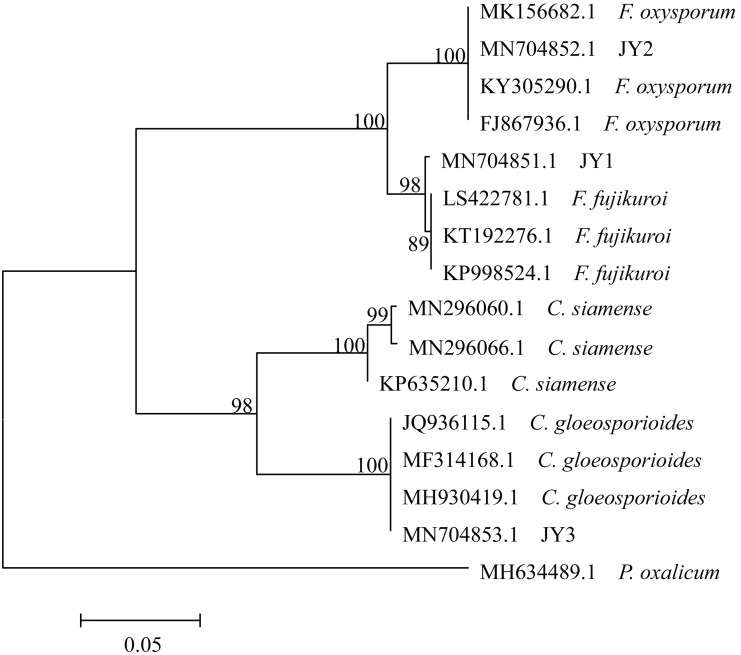
Phylogenetic tree of JY1, JY2 and JY3 as well as reference isolates. JY1, JY2 and JY3 were representative isolates used for identifying *F. fujikuroi, F. oxysporum* and *C. gloeosporioides.* The analysis involved 16 internal transcribed spacer sequences of nuclear ribosomal DNA. *Penicillium oxalicum* (MH634489.1) was used as an outgroup. The percentage of replicate trees in which the associated taxa clustered together in the bootstrap test (1,000 replicates) are shown next to the branches. The tree is drawn to scale, with branch lengths in the same units as those of the evolutionary distances used to infer the phylogenetic tree. The optimal tree with the sum of branch length = 0.57566860 is shown.

**Figure 2:**
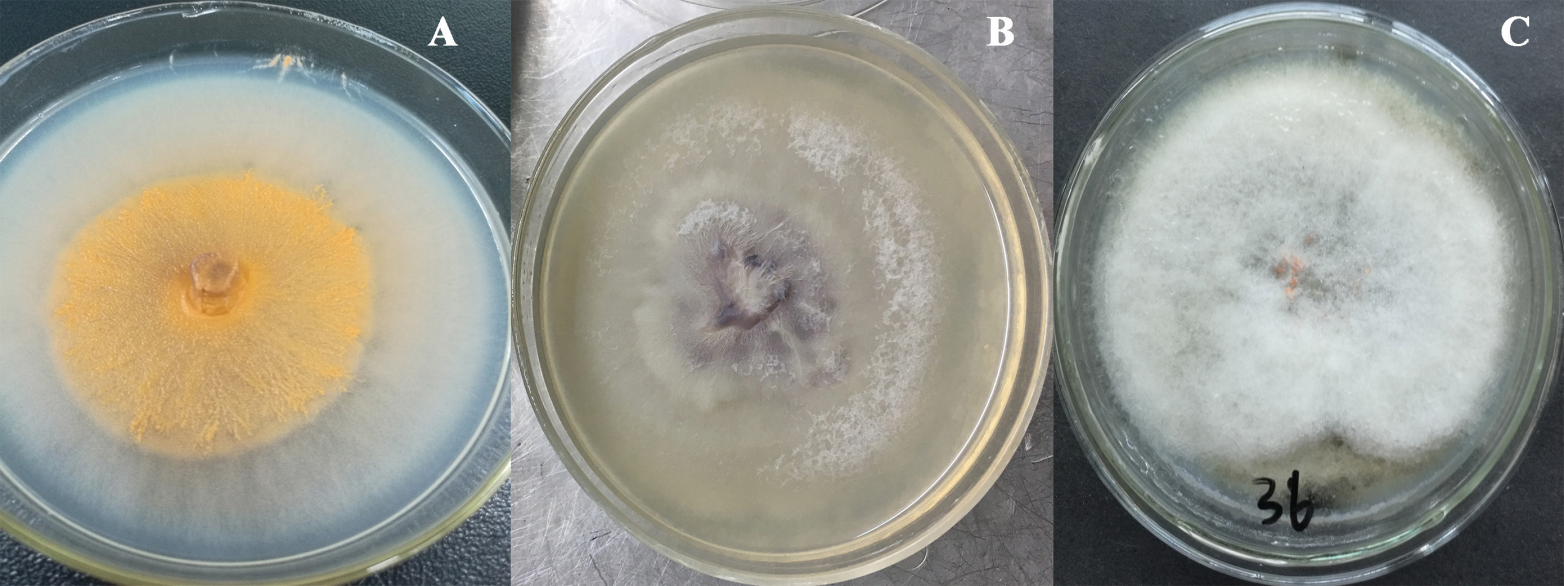
Fungal colonies of JY1, JY2 and JY3 on PDA. JY1 (A), JY2 (B) and JY3 (C) were representative isolates used for identifying *F. fujikuroi*, * F. oxysporum* and *C. gloeosporioides*. PDA, Potato dextrose agar.

The total size of ITS regions of JY1, JY2 and JY3 were 561, 557 and 588 bp, respectively. The phylogenetic analysis showed that the ITS sequences of JY1 and *F. fujikuroi* were clustered into the same group with 98% of bootstraps (Fig. 1). The ITS sequences of JY2 and JY3 were respectively clustered together with that of *F. oxysporum* and *C. gloeosporioides* into the same groups with 100% of bootstraps (Fig. 1). Morphologically, JY1 produced abundant orange sporodochia, the colony color was white to orange on PDA (Fig. 2A). The macroconidia were sparse, hyaline, sickle-shaped, with two to three septations, and measured 22.7 to 40.8 × 3.1 to 4.1 µm (*n* = 20). Microconidia were formed in chains or scattered, hyaline, aseptate or one septate. They were generally slender, clavate with a flattened base, and measured 5.2 to 15.0 × 1.5 to 3.8 (*n* = 20). Colony color of JY2 was white to purple on PDA (Fig. 2B). The macroconidia were sparse, hyaline, slightly sickle-shaped, with two septations, and measured 30.2 to 42.5 × 3.4 to 4.5 µm (*n* = 20). Microconidia were abundant, hyaline, aseptate, and formed abundantly in false heads. They were generally oval-ellipsoid to kidney-shaped and measured 5.2 to 15.0 × 1.5 to 3.8 (*n* = 20). Based on the morphological criteria presented in the *Fusarium* Laboratory Manual (Leslie & Summerell, 2006) and phylogenetic analysis (Fig. 1), JY1 and JY2 were identified as *F. fujikuroi* and *F. oxysporum*, respectively. Colony color of JY3 was white to pale grey with orange conidial masses near the inoculums point (Fig. 2C). The conidia were abundant and cylinder-shaped, and measured 9.0 to 14.7 × 3.1 to 4.1 µm (*n* = 20). These morphological characteristics were highly in agreement with a previous description of *C. gloeosporioides* (Rabha et al., 2016). Combining the morphological with phylogenetic analyses (Fig. 1), JY3 was identified as *C. gloeosporioides*.

### Occurrence rate of *F. fujikuroi* and *F. oxysporum* in the field

In the field, the occurrence rates of *F. fujikuroi* and *F. oxysporum* on the surfaces of healthy leaves did not vary amongst habitats occupied by *I. cairica*. *F. fujikuroi* and *F. oxysporum* always coexisted, and their occurrence rates were 100 ±0.00% on *I. cairica*.

### Comparison of *F. oxysporum* and *F. fujikuroi* abundance between infected and healthy leaves of *I. cairica* in the field

**Figure 3:**
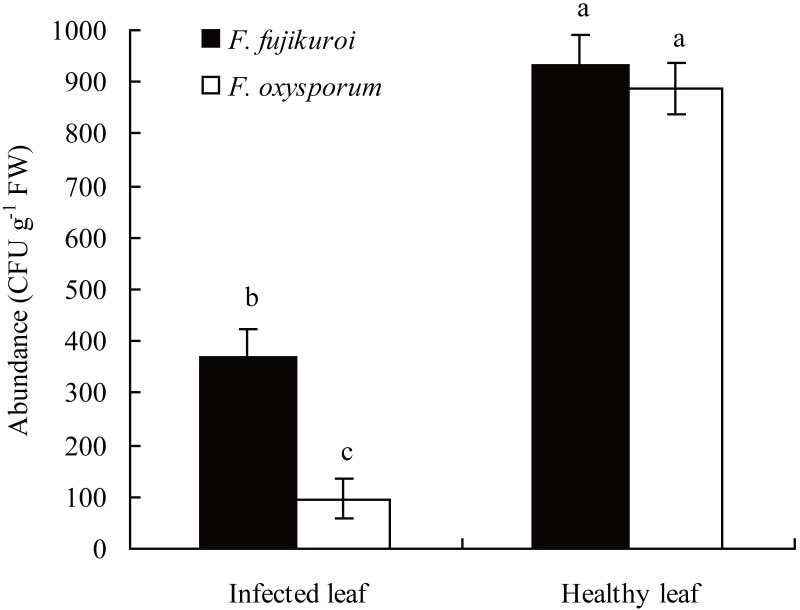
Comparison of *F. oxysporum* and *F. fujikuroi* abundance between infected and healthy leaves of *I. cairica*. The leaf infected naturally by *C. gloeosporioides* in the field was defined as infected leaf. Healthy leaf had no any disease symptom. Each value is the mean ± standard error of three replicates. Error bars indicate standard errors. Different letters above error bars indicate significant difference (*P* < 0.05) as determined by LSD test. CFU, colony-forming units; FW, fresh weight.

In the field, the abundance of *F. oxysporum* (*n* = 3, *P* = 0.000) and *F. fujikuroi* (*n* = 3, *P* = 0.000) on the surfaces of healthy leaves of *I. cairica* was significantly higher than that of *C. gloeosporioides* infected leaves, respectively (Fig. 3).

### The pathogenicity of *F. oxysporum* and *F. fujikuroi* to *I. cairica*

Inoculation with *F. oxysporum* and *F. fujikuroi* did not cause lesions on the leaves of *I. cairica*, whereas inoculation with *C. gloeosporioides* led to obvious infection symptom. The lesion area ratio caused by *C. gloeosporioides* was 2.11 ± 0.48% and significantly higher than that of inoculation with *F. oxysporum* (*n* = 8, *P* = 0.000) and *F. fujikuroi* (*n* = 8, *P* = 0.000) in addition to the negative control (*n* = 8, *P* = 0.000). The results showed that *F. oxysporum* and *F. fujikuroi* were non-pathogens of *I. cairica*.

### Effects of pre-inoculation with *F. oxysporum*, *F. fujikuroi* alone and a mixture of both on growth parameters of *I. cairica* infected with *C. gloeosporioides*

**Figure 4:**
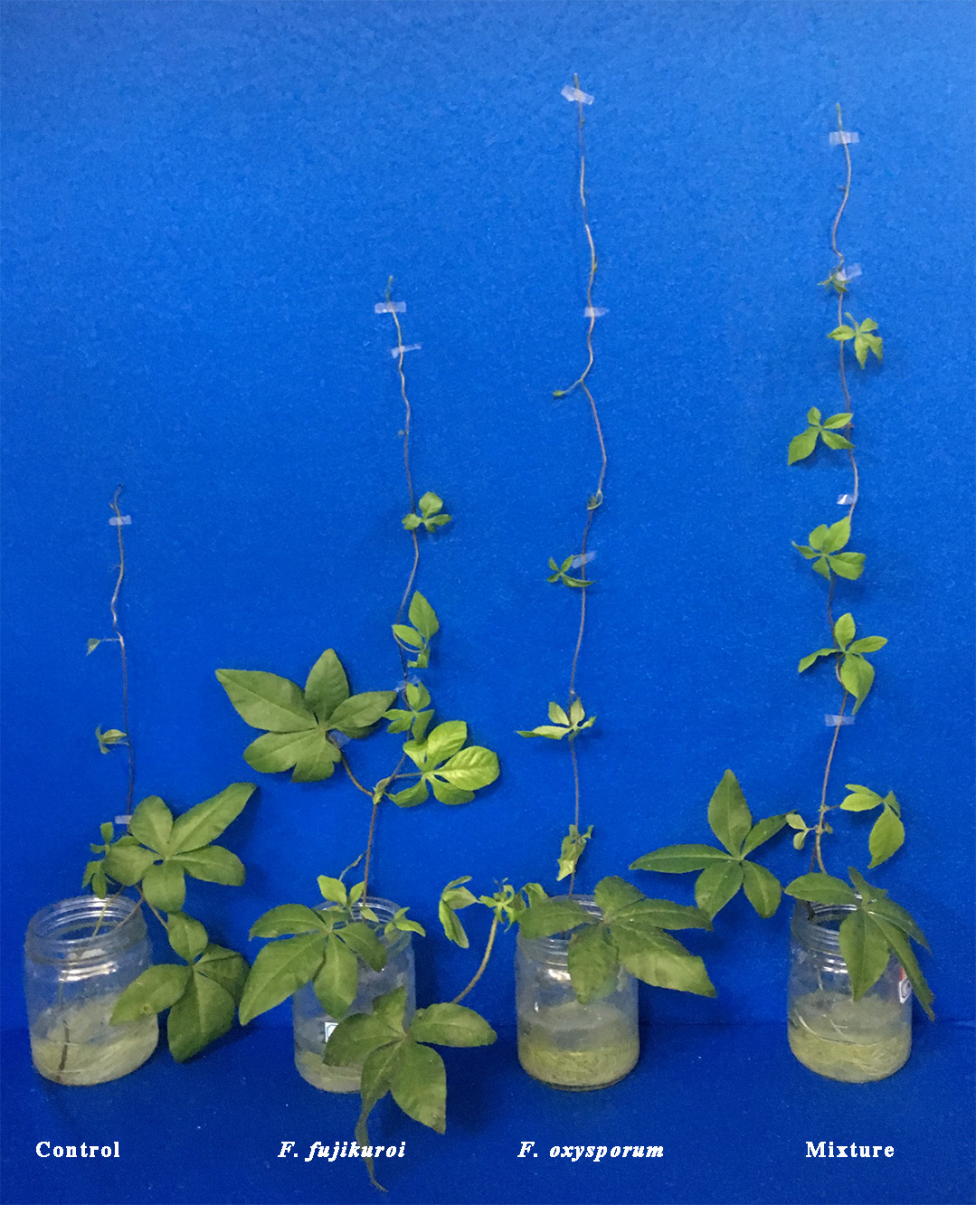
Growth responses of *I. cairica* inoculated with *F. oxysporum, F. fujikuroi* alone and a mixture of both to the infectionof *C. gloeosporioides*. The control group was sprayed with sterile potato dextrose broth.

**Figure 5:**
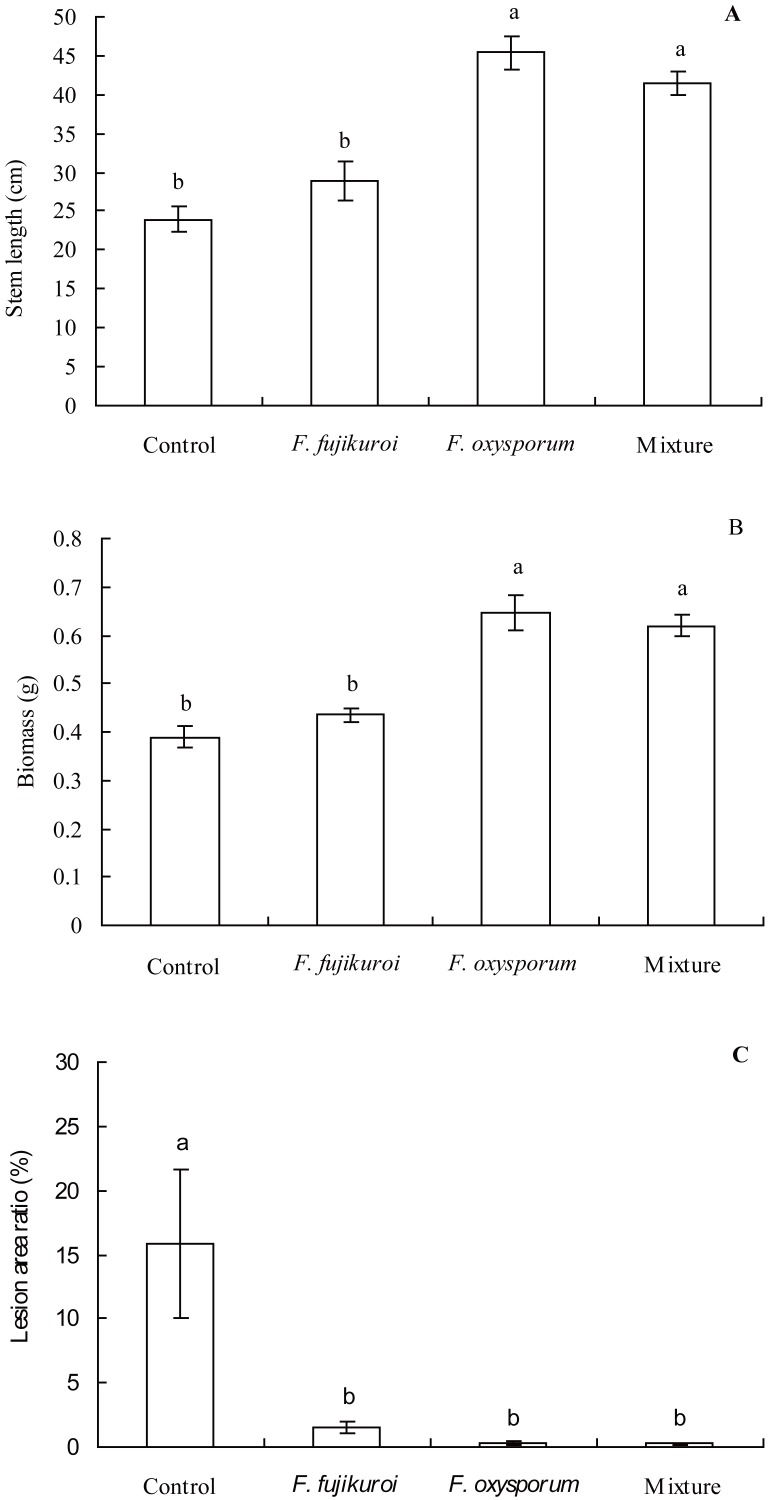
Effects of pre-inoculation with *F. oxysporum,F. fujikuroi* alone and a mixture of both on growth parameters of *I. cairica* infected with *C. gloeosporioides*. (A) Stem length. (B) Biomass. (C) Lesion area ratio. Each value is the mean ± standard error of five replicates per treatment. Error bars indicate standard errors. Different letters above error bars indicate significant difference (*P* < 0.05) as determined by LSD test.

The growth of *I. cairica* within treatment and control groups responded differentially to the infection of *C. gloeosporioides* (Fig. 4). There were significant differences in the leaf lesion area ratio (*df* = 3, 16, *F* = 6.863, *P* = 0.003; Fig. 5C), stem length (*df* = 3, 16, *F* = 25.633, *P* = 0.000; Fig. 5A) and biomass (*df* = 3, 16, *F* = 25.129, *P* = 0.000; Fig. 5B) of *I. cairica* within treatment and control groups. Compared to the control, pre-inoculation with *F. oxysporum* (*n* = 5, *P* = 0.02), *F. fujikuroi* (*n* = 5, *P* = 0.03) alone and in a mixture (*n* = 5, *P* = 0.02) significantly reduced leaf lesion area ratio of *I. cairica* plants caused by *C. gloeosporioides* (Fig. 5C). Moreover, pre-inoculation with *F. oxysporum* and mixture of both fungi significantly increased the stem length (*F. oxysporum*: *n* = 5, *P* = 0.000; mixture of both fungi: *n* = 5, *P* = 0.000; Fig. 5A) and biomass (*F. oxysporum*: *n* = 5, *P* = 0.000; mixture of both fungi: *n* = 5, *P* = 0.000; Fig. 5B) of *I. cairica* plants. However, the two growth parameters were not promoted by pre-inoculation with *F. fujikuroi* (biomass: *n* = 5, *P* = 0.220, Fig. 5B; stem length: *n* = 5, *P* = 0.103, Fig. 5A), and significantly lower than that of *I. cairica* plants inoculated with *F. oxysporum* (biomass: *n* = 5, *P* = 0.000, Fig. 5B; stem length: *n* = 5, *P* = 0.000, Fig. 5A) and mixture of both fungi (biomass: *n* = 5, *P* = 0.000, Fig. 5B; stem length: *n* = 5, *P* = 0.000, Fig. 5A).

### Effects of pre-inoculation with *F. oxysporum***,*F. fujikuroi*alone and a mixture of both on physiological characteristics of*I. cairica*infected with*C. gloeosporioides***

**Figure 6:**
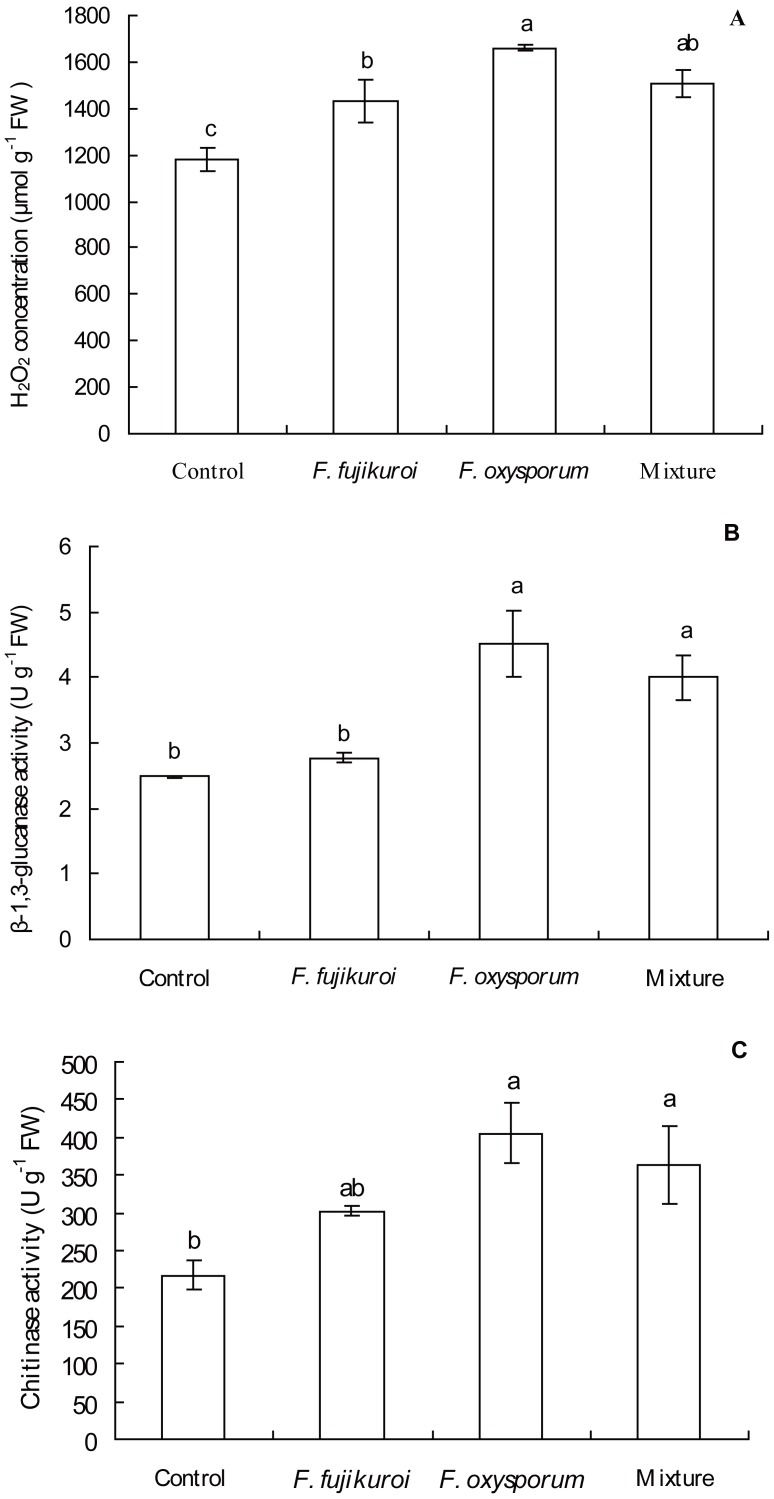
Effects of pre-inoculation with *F. oxysporum*, *F. fujikuroi* alone and a mixture of both on physiological characteristics of *I. cairica* infected with *C. gloeosporioides*. (A) Hydrogen peroxide (H_2_O_2_) concentration. (B) β-1,3-glucanase activity. (C) Chitinase activity*.* Each value is the mean ± standard error of four replicates per treatment. Error bars indicate standard errors. Different letters above error bars indicate significant difference (*P* < 0.05) as determined by LSD test. FW: Fresh weight.

There were significant differences in H_2_O_2_ concentration (*df* = 3, 12, *F* = 11.025, *P* = 0.001; Fig. 6A), β-1,3-glucanase (*df* = 3, 12, *F* = 10.318, *P* = 0.001; Fig. 6B) and chitinase activities (*df* = 3, 12, *F* = 5.650, *P* = 0.012; Fig. 6C) in leaves of *I. cairica* within treatment and control groups. *F. oxysporum* (*n* = 4, *P* = 0.000), *F. fujikuroi* (*n* = 4, *P* = 0.011) alone and as a mixture (*n* = 4, *P* = 0.002) significantly increased H_2_O_2_ concentration in leaves of *I. cairica* plants infected with *C. gloeosporioides*, compared with the control (Fig. 6A). Furthermore, pre-inoculation with *F. oxysporum* and mixture of both fungi significantly improved β-1,3-glucanase (*F. oxysporum*: *n* = 4, *P* = 0.000; mixture of both fungi: *n* = 4, *P* = 0.004; Fig. 6B) and chitinase activities (*F. oxysporum*: *n* = 4, *P* = 0.002; mixture of both fungi: *n* = 4, *P* = 0.011; Fig. 6C) in leaves, whereas pre-inoculation with *F. fujikuroi* did not enhance the two enzymes activities (β-1,3-glucanase activity: *n* = 4, *P* = 0.511, Fig. 6B; chitinase activity: *n* = 4, *P* = 0.106, Fig. 6C). β-1,3-glucanase activity (Fig. 6B) in leaves pre-inoculated with *F. fujikuroi* was significantly lower than that in leaves pre-inoculated with *F. oxysporum* (*n* = 4, *P* = 0.002) and mixture of both fungi (*n* = 4, *P* = 0.014).

### Effects of pre-inoculation with *F. oxysporum*, *F. fujikuroi* alone and a mixture of both on hormone content and *NPR1* expression in leaves of *I. cairica* infected with *C. gloeosporioides*

**Figure 7:**
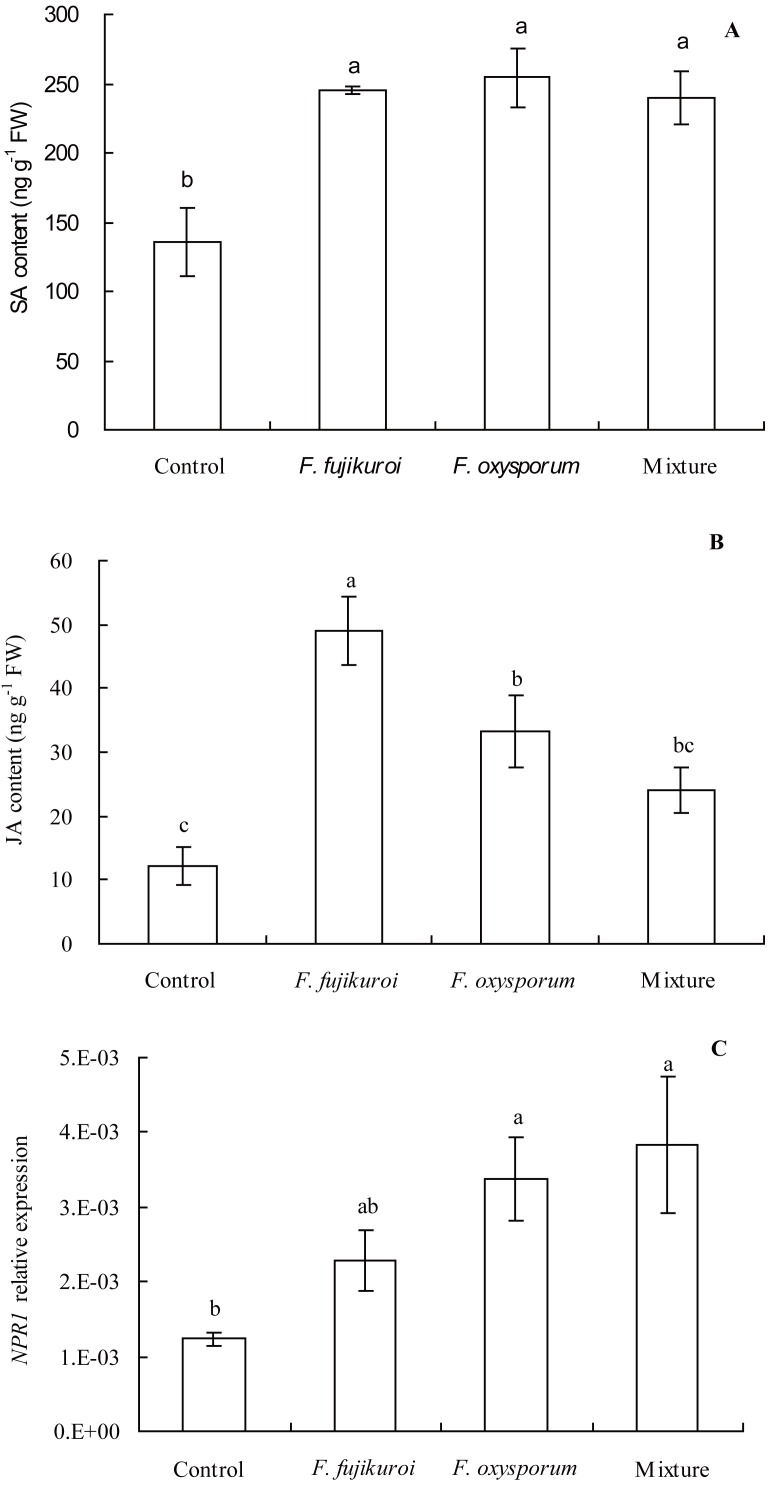
Effects of pre-inoculation with *F. oxysporum F. fujikuroi* alone and a mixture of both on hormone content and *NPR1* expression in leaves of *I. cairica* infected with *C. gloeosporioides*. (A) Salicylic acid (SA) content. (B) Jasmonic acid (JA) content. (C) Non-expressor of pathogenesis-related genes-1 (*NPR1*) expression*.* Each value is the mean ± standard error of three replicates per treatment. Error bars indicate standard errors. Different letters above error bars indicate significant difference (*P* < 0.05) as determined by LSD test. FW, fresh weight.

There were significant differences in *NPR1* expression (*df* = 3, 8, *F* = 4.112, *P* = 0.049; Fig. 7C), SA (*df* = 3, 8, *F* = 8.637, *P* = 0.007; Fig. 7A) and JA content (*df* = 3, 8, *F* = 11.751, *P* = 0.003; Fig. 7B) in leaves of *I. cairica* within treatment and control groups. In contrast with the control, pre-inoculation with *F. oxysporum* (*n* = 3, *P* = 0.002), *F. fujikuroi* (*n* = 3, *P* = 0.004) alone and a mixture of both (*n* = 3, *P* = 0.005) significantly elevated SA content in leaves of *I. cairica* plants infected with *C. gloeosporioides* (Fig. 7A). Moreover, except for pre-inoculation with mixture, pre-inoculation with *F. oxysporum* (*n* = 3, *P* = 0.011) and *F. fujikuroi* (*n* = 3, *P* = 0.000) significantly enhanced JA content in leaves (Fig. 7B). JA content in leaves pre-inoculated with *F. fujikuroi* was significantly higher than that in leaves pre-inoculated with *F. oxysporum* (*n* = 3, *P* = 0.038) and mixture of both fungi (*n* = 3, *P* = 0.005). Furthermore, in contrast with the control, pre-inoculation with *F. oxysporum* (*n* = 3, *P* = 0.030) or a mixture of both fungi (*n* = 3, *P* = 0.012) significantly increased *NPR1* expression of leaves infected with *C. gloeosporioides* (Fig. 7C). However, there was no significant upgrade following pre-inoculation with *F. fujikuroi* in *NPR1* expression (*n* = 3, *P* = 0.232, Fig. 7C).

## Discussion

### The occurrence, abundance and pathogen resistance attributes of *F. oxysporum***and***F. fujikuroi*

Symbiosis between plants and microbes is a very common ecological relationship. Host plants obtain diverse benefits from the symbiosis involving the improvement of nutrition availability (Ngwene et al., 2016;Bertolazi et al., 2019), yields (Xia et al., 2016) and tolerance against abiotic as well as biotic stresses (Daneshkhah, Grundler & Wieczorek, 2018; Song et al., 2015). In this study, the occurrence rates of *F. oxysporum* and *F. fujikuroi* on leaf surface of *I. cairica* reached up to 100% regardless of habitat. The results indicated that the symbiosis between *F. oxysporum*, *F. fujikuroi* and *I. cairica* was established naturally in habitats and was considerably stable*.* Moreover, with artificial inoculation, we found that *F. oxysporum* and *F. fujikuroi* were not pathogenic to *I. cairica*, conversely, they enhanced pathogen resistance of *I. cairica* against *C. gloeosporioides* and significantly reduced lesion area ratio of leaves (Fig. 5C)*.* Previous studies have shown that pathogen resistance of plants can be induced by *F. oxysporum* and *F. fujikuroi* (Patil et al., 2011; Veloso & Díaz, 2012). Interestingly, under natural conditions, the abundance of *F. oxysporum* and *F. fujikuroi* on healthy leaves was significantly higher than that of *C. gloeosporioides* infected leaves (Fig. 3)*.* The results further suggested that the health of *I. cairica* plants was relevant to high abundance of symbiotic *F. oxysporum* and *F. fujikuroi*. Since *I. cairica* first invaded Hong Kong as an exotic species in 1912 (Yuan et al., 2019), it has experienced an invasive history spanning 100 years in China. Saikkonen et al. (2016) reported that symbiosis was the outcome of long-term co-evolution between microbes and host plants. To our knowledge, the symbiosis between microbes and *I. cairica* was first reported in the present study. Thus, it is not clear whether the symbiosis between *F. oxysporum*, *F. fujikuroi* and *I. cairica* is inherent in its native location or established afterwards *via* co-evolution in the invasive regions. We believe that, by investigating the presence or absence of *F. oxysporum* and *F. fujikuroi* on *I. cairica* in its native locations, the origin of their symbiosis can be better understood. If *I. cairica* in its native locations harbors *F. oxysporum* and *F. fujikuroi*, we can conclude that their symbiosis is inherent. If not, then it is likely that their symbiosis is established in invasive regions of *I. cairica*. Nevertheless, our findings suggested that the symbiosis had important ecological significance in alleviating the pathogen pressure of *C. gloeosporioides* imposed on *I. cairica* in nature.

### Physiological mechanism of pathogen resistance induced by *F. oxysporum***and***F. fujikuroi*

H_2_O_2_ was used to measure ROS. When plants are attacked by pathogens, hypersensitive responses will be elicited and H_2_O_2_ will be accumulated in plants (Lin & Ishii, 2009). As H_2_O_2_ may directly kill pathogens at infection sites (Lin & Ishii, 2009), we inferred that significant increase of H_2_O_2_ concentration in leaves of *I. cairica* induced by *F. oxysporum*, *F. fujikuroi* alone and a mixture of both fungi (Fig. 6A) might have strengthened inhibitory effects on *C. gloeosporioides* at infection sites and prevented further expansion of leaf lesion, resulting in significantly less lesion area ratio relative to the control (Fig. 5C). In addition, H_2_O_2_ also can be employed as a signal molecule to mediate the levels of downstream signal of SA and JA (Ren & Dai, 2012) and induces pathogen resistance of plants (Keshavarz-Tohid et al., 2016; Deng et al., 2016). In our study, significantly increased SA and JA content (Figs. 7A and 7B) in leaves of *I. cairica* pre-inoculated with *F. oxysporum*, *F. fujikuroi* alone and mixture should be relevant to H_2_O_2_ accumulation in leaves (Fig. 6A).

In plants, SA- or JA-dependent defense responses are generally activated by non-pathogens and pathogens with different lifestyles, such as biotrophy and necrotrophy (Paparu et al., 2007; Chen et al., 2018). SA and JA are important signaling molecules in plant defense responses. Through signaling transduction, SA and JA signaling mediates *NPR1* expression (Stein et al., 2008; Nic-Matos et al., 2017; Ali et al., 2017), further eliciting distinct sets of resistance gene expression. SA signaling involves *PR* genes encoding PR proteins including β-1,3-glucanase and chitinase (Stein et al., 2008). JA signaling involves some genes encoding defense-related proteins, such as defensin (Tiwari et al., 2017; Sarkar, Jana & Sikdar, 2017; Brown et al., 2003). Previous studies have shown that non-pathogenic *F. oxysporum* and *F. fujikuroi* induce up-regulation of *PR1* genes expression (Veloso & Díaz, 2012) and activities of chitinase and β-1,3-glucanase (Fuchs, Moënne-Loccoz & Défago, 1997; Patil et al., 2011) and improve pathogen resistance of host plants. However, our results showed that, in contrast to the control, although *F. oxysporum* and *F. fujikuroi* alone and in mixture induced significantly higher SA content in leaves of *I. cairica* (Fig. 7A), the transmission efficiency of SA signaling between the three treatments was largely different. Colonization by *F. fujikuroi* failed to transmit SA signaling and did not up-regulate *NPR1* expression (Fig. 7C), chitinase and β-1,3-glucanase activities (Figs. 6B and 6C), whereas colonization by *F. oxysporum* and the mixture of both fungi successfully transmitted SA signaling, significantly up-regulated *NPR1* expression (Fig. 7C), chitinase and β-1,3-glucanase activities (Figs. 6B and 6C). Interestingly, compared with *F. oxysporum* and the mixture of both fungi, *F. fujikuroi* induced significantly higher JA content but significantly lower β-1,3-glucanase activity in leaves of *I. cairica* plants (Figs. 6B and 7B). The results showed that excessive JA content in *I. cairica* plants induced with *F. fujikuroi* antagonized SA signaling defense pathway and suppressed SA-dependent β-1,3-glucanase activity. Previous studies have suggested JA signaling cross-talk with SA signaling defense pathways *via NPR1* (Spoel et al., 2003; Withers & Dong, 2016) antagonizes SA signaling and suppresses SA-dependent genes expression (Kachroo et al., 2001). Therefore, our findings showed that *F. oxysporum* played a dominant role in inducing pathogen resistance of *I. cairica* against *C. gloeosporioides* because its presence alone or coexistence with *F. fujikuroi* alleviated the antagonism of JA signaling on SA-dependent β-1,3-glucanase activity.

It is well known that β-1,3-glucanase inhibit fungal growth through degrading glucan in the cell wall of pathogenic fungi (Balasubramanian et al., 2012; Vieira et al., 2010; Li et al., 2015). In our study, compared to the control and *F. fujikuroi* treatment, with higher β-1,3-glucanase activities (Fig. 6B), *I. cairica* plants induced with *F. oxysporum* and a mixture of both fungi strengthened pathogen resistance against *C. gloeosporioides* and achieved greater stem length and biomass (Figs. 5A and 5B).

## Conclusions

In natural habitats, healthy leaves of *I. cairica* plants established stable symbiosis with non-pathogenic *F. fujikuroi* and *F. oxysporum* and had a higher abundance of the both fungi relative to *C. gloeosporioides* infected leaves. Although *F. fujikuroi* and *F. oxysporum* could induce pathogen resistance of *I. cairica* against *C. gloeosporioides*, *F. oxysporum* played a dominant role in inducing pathogen resistance. Its presence alleviated the antagonism of JA on the SA signaling defense pathway and enabled *I. cairica* plants to maintain relatively higher level of resistance against *C. gloeosporioides.*

The interactions between plants and symbiotic microbes have been well studied in plant invasion ecology (Soares et al., 2016; Shearin et al., 2018). Some microbial symbionts have been identified as drivers in successful plant invasions owing to their plant growth promoting effects (Dai et al., 2016). The results obtained in the present study provide new evidence that epiphytic *F. fujikuroi* and *F. oxysporum* act as pathogen resistance inducers of the invasive plant *I. cairica*. However, in this study, we only targeted the selected epiphytic *F. fujikuroi* and *F. oxysporum* to explore their contributions of pathogen resistance to *I. cairica*, which might have overlooked other microbial symbionts associated with pathogen resistance against *C. gloeosporioides*. Therefore, future works should systematically investigate the overall symbiotic microbial community (endophytes and epiphytes) of *I. cairica*, screen microbial species functioning as plant pathogen resistance inducers, and thereby extend the study of ecological and physiological mechanisms inducing pathogen resistance. In addition, in relation to the management and control of *I. cairica*, the disruption of the symbiosis between *I. cairica* and mutualistic microbes might provide a potentially effective strategy.

##  Supplemental Information

10.7717/peerj.8889/supp-1 Supplemental Information 1 Raw data The occurrence rate of *F. oxysporum* and *F. fujikuroi* on *I. cairica* in different habitats. The comparison of the abundance of *F. oxysporum* and *F. fujikuroi* between infected and healthy leaves of *I. cairica* in the field. The pathogenicity of *F. oxysporum* and *F. fujikuroi* to *I. cairica*. The effects of pre-inoculation with *F. oxysporum*, *F. fujikuroi* alone and a mixture of both on growth parameters of* I. cairica* infected with *C. gloeosporioides*. The effects of pre-inoculation with *F. oxysporum*, *F. fujikuroi* alone and a mixture of both on physiological characteristics of *I. cairica* infected with *C. gloeosporioides*. The effects of pre-inoculation with *F. oxysporum*, *F. fujikuroi* alone and a mixture of both on hormone content and *NPR1* expression in leaves of *I. cairica*. The morphological characteristic of representative isolates JY1, JY2 and JY3.

## Additional Information and Declarations

### DNA Deposition

The following information was supplied regarding the deposition of DNA sequences: The ITS sequences of representative isolates JY1, JY2 and JY3 are available at GenBank: MN704851.1, MN704852.1 and MN704853.1.

### Data Availability

The following information was supplied regarding data availability: The raw data measurements are available in the Supplemental Files.
